# Conjugation of gum Arabic and lentil protein hydrolysates through Maillard reaction: Antioxidant activity, volatile compounds, functional and sensory properties

**DOI:** 10.1002/fsn3.3966

**Published:** 2024-01-15

**Authors:** Amir Rezvankhah, Babak Ghanbarzadeh, Homaira Mirzaee, Ali Ahmadi Hassan Abad, Ali Tavakkoli, Alireza Yarmand

**Affiliations:** ^1^ Department of Food Science and Technology, Razi Food Chemistry Lab, College of Agriculture and Natural Resources University of Tehran Tehran Iran; ^2^ Department of Food Science and Technology, Faculty of Agriculture University of Tabriz Tabriz Iran; ^3^ Department of Food Science and Technology, Faculty of Agriculture Tarbiat Modares University Tehran Iran; ^4^ Applied Science Learning Center Sham Sham Food Science Group Shiraz Iran; ^5^ Student of internal diseases of large animals, Veterinary Faculty of Research Science Unit Islamic Azad University Tehran Iran

**Keywords:** lentil protein hydrolysates, maillard grafting, umami taste, volatile compounds

## Abstract

Lentil protein hydrolysates (LPH) and lentil protein hydrolysates cross‐linked (LPHC) were grafted with gum Arabic (GA) through a wet Maillard reaction at 100°C for 2 h and called MLPH and MLPHC. The samples were assessed for absorption, degree of grafting (DG), surface hydrophobicity, antioxidant activity, molecular weight (MW) profile, chemical alteration, volatile compounds, functional and sensory properties. Results showed that Maillard grafting led to increase in absorption and DG (maximum value: MLPHC), and led to the reduction of the surface hydrophobicity and antioxidant activity (minimum value: MLPHC). MW profiles indicated that MLPH and MLPHC formed new bands at MW >250 kDa. Regarding the Fourier transform infrared spectroscopy (FTIR), Maillard conjugation led to the occurrence of peaks at 1759 and 1765 cm^−1^, while the intensities of amide I bands at 1637 and 1659 cm^−1^ and amide II bands at 1498 and 1495 cm^−1^ were decreased. Hydrolysis, cross‐linking, and especially Maillard grafting provided well‐balanced content of volatile components. Indeed, the proportions of alcohols, ketones, aldehydes, and acids were changed, thereby, the inherent grassy and planty tastes were diminished while new umami taste was developed. Maillard grafting led to significant improvement of functional properties, while MLPH and MLPHC indicated the highest emulsifying activity at pH 10.0 (73.76 and 70.12 m^2^/g, respectively) and stability (369.64 and 288.22 min), foaming capacity (88.57% and 142.86%) and stability (60.57% and 72%). Sensory analysis has demonstrated that umami taste was highly developed in MLPH and MLPHC, which can be well considered as meat proteins and flavor enhancers such as monosodium glutamate (MSG).

## Graphical abstract

The lentil protein hydrolysates (LPH) cross‐linked via transglutaminase and conjugated through Maillard reaction to produce a substitute for monosodium glutamate (MSG).

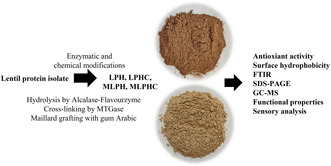

## 1 INTRODUCTION

The world population is rapidly growing, which has been predicted to reach 9.7 billion by 2050 (Zhang et al., 2022; Zhao et al., 2022). This can ultimately change the quantity and also the quality of human diet. Thus, the necessity to produce more sustainable food products has been increased. Besides, the production of more sustainable animal‐based food products has been converted to a concerning issue. Therefore, research on the development of plant‐based food products that are in high demand has been increased (Mirzaee et al., 2023; Zhang et al., 2022). Moreover, being vegetarian (having vegan diet) has attained high popularity, which has led to the consumption of more plant‐based products. For instance, Australians (2.5 million, 12.1%) are consuming diets that are composed of entirely or primarily plant origin. Therefore, a great research field has been formed to develop plant‐based products (Rezvankhah et al., 2023; Zhang et al., 2022).

Recently, attention to the pulse crops has been increased due to high sustainability, resistance to climate change, and also high‐quality protein sources (Bessada et al., 2019; Rezvankhah et al., 2021a). Pulses, such as peas, common beans, cowpea, chickpea, fava beans, lupin, and lentil, contain high concentrations of proteins composed of globulins and albumins, providing about 33% of dietary needs (Bessada et al., 2019; Rezvankhah, Yarmand, & Ghanbarzadeh, 2022). Lentil protein (21%–31% of total seed) is primarily composed of storage proteins, mainly salt‐soluble globulins and water‐soluble albumins (Rezvankhah et al., 2021a, 2021b). Lentil protein has been recognized to contain high amounts of essential amino acids such as lysine and leucine, compared to soy protein (Mirzaee et al., 2023; Rezvankhah et al., 2023). Several studies have shown that lentil protein has high nutritional and biological quality along with favorable functional properties (Avramenko et al., 2013; Chang et al., 2015; Joshi et al., 2012; Karaca et al., 2013; Rezvankhah et al., 2021b; Rezvankhah, Yarmand, & Ghanbarzadeh, 2022). However, similar to other plant proteins such as pea protein, the lentil protein encounters low solubility due to having a high content of hydrophobic amino acids. Its surface charge is also low that limits its utilization as an emulsifier, which attenuates the functionality of protein (Rezvankhah et al., 2021b; Zhang et al., 2022). Up to date, several routes have been applied to modify the lentil protein's biological and functional properties. Accordingly, physical (ultrasound and high pressure) and enzymatic (hydrolysis and cross‐linking) methods have been individually or combinatorially investigated to modify the protein both biologically and functionally (Garcia‐Mora et al., 2015; Gharibzahedi & Smith, 2021; Rezvankhah, Yarmand, & Ghanbarzadeh, 2022). Despite this, there are not enough data regarding the chemical modification of lentil protein, especially through Maillard reaction.

Maillard reaction is a chemical process that usually occurs through protein–carbohydrate conjugation, which is considered as the most straightforward and common method to modify proteins (Zha, Yang, et al., 2019). The interaction conventionally occurs between the terminal reducing group of the carbonyl located on carbohydrate and the ɛ‐amino group of amino acids (mostly lysine, glutamine, etc.) located on the proteins. Several studies have reported that Maillard reaction can produce glycopeptides with improved solubility, thermal stability, and emulsifying properties (Ke & Li, 2023; Zhang et al., 2022; Zhao et al., 2022). Moreover, Maillard reaction can lead to the development of meaty‐analog flavor and mask the unpleasant odor and flavor of plant proteins (Song et al., 2013; Wei et al., 2018). Especially, the produced peptides from plant proteins have a high potential in the generation of meaty flavor precursors (Song et al., 2013; Sun et al., 2022).

Conjugation or Maillard reaction is commonly performed between a protein and a carbohydrate through a dry‐heating condition (Naik et al., 2021; Zha, Yang, et al., 2019). The mixture of protein and carbohydrate (at a certain ratio) is dried using freeze‐drying and becomes powder. Then, to facilitate the conjugation reaction, the obtained powder is subjected to controlled heating condition (around 50–70°C), specific relative humidity (around 65%–79%), and storage days ranged between 1 and 14 (Zha, Yang, et al., 2019; Zhao et al., 2022). Despite this, it is more recommended in laboratory scale and is limited in commercial application, likely due to the prolonged reaction time and uncontrollable progress or reaction level (Naik et al., 2021; Zhang et al., 2022). On the other side, the wet‐heating process in terms of Maillard reaction has been introduced to be more promising since the contact and subsequently conjugation of carbohydrate and protein are maximized in the aqueous solution, leading to reduction of reaction time (Zhang et al., 2022; Zhao et al., 2022).

Maillard reaction has been divided into three stages, including initial stage, intermediate stage, and advanced stage (Zhang et al., 2022; Zhao et al., 2022). The initial stage is well known to Schiff‐base formation so that the reducing carbonyl group of carbohydrate is reacted with free ɛ‐amino group of amino acids/peptides/proteins. In this regard, the Schiff base is then converted into the colorless Amadori compounds, implying the occurrence of the initial stage of Maillard reaction. By proceeding with the reaction, the intermediate compounds are degraded through the condensation with free amino acids. In this stage, Strecker aldehydes are generated (Zhang et al., 2022; Zhao et al., 2022). Several degradation reactions may occur and Amadori compounds are degraded at prolonged process time, calling advanced stage. Regarding this, advanced glycation end products (AGEs), such as pentosidine and N(ε)‐Carboxymethyl lysine, are generated (Zhang et al., 2022; Zhao et al., 2022). The advanced stage leads to the formation of brownish color specified to the melanoidins, implying the occurrence of the advanced stage of Maillard reaction (Zhang et al., 2022; Zhao et al., 2022). However, AGEs have been shown to exert some detrimental impacts on human health. Regarding this, the inflammation issues, oxidative stress, chronic diabetes, and cardiovascular diseases can occur. Hence, it seems so crucial to recognize the Maillard reaction stage. Especially, the Maillard reaction can be controlled within initial or intermediate stage to avoid the formation of advanced compounds.

Maillard reaction can not only lead to improvement of functional properties of plant proteins, but can also lead to formation of flavor originated from the volatile components produced from intermediate stage (Zha, Dong, et al., 2019). Such volatile components are aldehydes, ketones, acids, and esters (Zha, Dong, et al., 2019; Zha, Yang, et al., 2019). The main flavors that have been produced are umami, meaty, and kokumi, which provide rational foundation for tailoring the analog flavors (Yan et al., 2021; Zha, Yang, et al., 2019). Especially, the findings of the previous reports have demonstrated that initial hydrolysis of proteins and subsequent conjugation of produced peptides with carbohydrates can potentially lead to the formation of meaty and umami flavors (Chiang et al., 2022; Liu et al., 2012; Wei et al., 2018; Yan et al., 2021; Zhou et al., 2021). Moreover, the previous achievements have shown that bitterness of plant protein hydrolysates can be reduced through the Maillard reaction while the umami taste was enhanced (Song et al., 2013; Zhou et al., 2021). Thus, sensorial taste of plant protein hydrolysates can be improved through the conjugation between amino acids/peptides/proteins and carbohydrates or reducing sugars. Maillard reaction intermediates would contribute to the improved test, mostly derived from enzymatic hydrolysates of plant proteins. The enzymatic hydrolysis of proteins can release specific amino acids or peptides, enhancing the meat analog flavor or umami taste when the produced peptides are conjugated with reducing sugars or carbohydrates (Song et al., 2013; Zhou et al., 2021). In addition, Maillard products can be substituted with flavor enhancers such as monosodium glutamate (MSG) with diverse health risks (Kesherwani et al., 2022; Zanfirescu et al., 2019).

To the best of our knowledge, no advanced research was accomplished regarding the Maillard reaction between the lentil protein hydrolysates and gum Arabic (GA). The produced conjugates were investigated for surface hydrophobicity, molecular weight profile, antioxidant activity, chemical alteration, volatile compounds, functional properties, and sensory analysis.

## 2 MATERIALS AND METHODS

### 2.1 Materials

Lupine protein isolate (LPI) with protein content of 85% was produced according to the alkalization and precipitation method (Rezvankhah et al., 2021a). Alcalase as an endopeptidase (the commercially called Alcalase 2.4 L), company reported activity of 2.4 anson units per gram (AU/g) with a density of 1.18 g/mL that has been originated from *Bacillus licheniformis*, and Flavourzyme as an exopeptidase (the commercially called Flavourzyme 1000 L), company reported activity of 1000 leucine amino peptidase units per gram (LAPU/g) with a density of 1.28 g/mL that has been originated from *Aspergillus oryzae* were provided from Novozymes Co. (Bagsværd outside of Copenhagen). Alcalase and Flavourzyme were used to produce peptides or single amino acids. Microbial transglutaminase (MTGase) with the activity of 100 U/g was prepared from Ajinomoto Co. Gum Arabic (GA) was provided from Ingredion Co. Trinitrobenzenesulfonic acid (TNBS) was purchased from Sigma Company. All other chemicals used were of analytical and HPLC (high performance liquid chromatography) grade.

### 2.2 Hydrolysis of LPI

Initially, the LPI solution was provided at 3% (w/w), followed by the denaturation of protein at 90°C for 30 min. In the next stage, the protein sequentially underwent the hydrolysis process using Alcalase and Flavourzyme (Rezvankhah et al., 2023). The hydrolysis was carried out at the enzyme‐to‐substrate (E:S) ratio of 2:100, and the optimal activity of enzymes was carried out during which the temperatures were 60 and 50°C and the pH values were 8.0 and 7.0 for Alcalase and Flavourzyme, respectively (based on the suggestions rendered by the respective enzyme producers). The enzymatic reaction was initiated using Alcalase to reach a degree of hydrolysis (DH) of 17% which took 120 min, then the enzyme was inactivated by heating at 90°C for 10 min and the solution was cooled to 50°C and the pH was set at 7.0, followed by the addition of Flavourzyme to proceed with the hydrolysis for further 60 min, reaching a DH value of 36% (Rezvankhah et al., 2023). The Flavourzyme was also inactivated at the same condition, followed by centrifuging the peptide solution at 15000 × *g* for 15 min to separate large polypeptides which had not undergone the hydrolysis action. The peptide solution (supernatant) was converted into powder through a spray‐drying process at the inlet and outlet temperatures of 170 and 85°C with the compressor pressure of 0.35–0.42 MPa (Rezvankhah et al., 2021b). The produced hydrolysate powder was collected and named lentil protein hydrolysate (LPH).

### 2.3 Cross‐linking of LPH

Initially, the LPH solution was prepared at 10% (w/w), followed by providing the optimum conditions of MTGase. On this basis, the LPH solution was heated and the temperature and pH were set at 45°C and 8.0, respectively, followed by the addition of MTGase at E:S of 1.2% (w/w), and treatment was performed for 5 h (Rezvankhah et al., 2023). After enzymatic treatment, the enzyme was terminated by heating the solution for 10 min at 90°C, followed by centrifuging at 11300 × *g* for 10 min at the ambient temperature. The collected supernatant was spray‐dried at the above‐mentioned conditions. The powdered solution was termed lentil protein hydrolysate cross‐linked (LPHC) and stored at 4°C for further experiments.

### 2.4 Conjugation of LPH and LPHC with GA

Glycopeptide was synthesized according to the method described by Zha, Dong, et al., 2019; Zha, Yang, et al., 2019 with some modifications. Briefly, produced hydrolysates, including LPH and LPHC, were mixed with GA at a proportion of 1:4 (w/w), and then hydrated in double‐distilled water to reach a solution of 10% w/w. The hydration of hydrolysates and GA proceeded overnight using a magnetic stirrer set at 500 revolutions per minute (rpm) (room temperature). The well‐hydrated mixtures were prepared for Maillard reaction by adjusting the pH at 9.0, then mixture solutions of hydrolysates and GA were subjected to thermal treatment at 100°C for 2 h using an oil bath and the same stirring condition. Then, the solution was cooled down to 35°C and the pH was adjusted to 7.0, followed by converting it into powder through a spray‐drying process at the above‐mentioned conditions. The Maillard‐treated LPH and LPHC were named “MLPH” and “MLPHC,” implying the conjugated LPH and LPHC with GA, respectively.

### 2.5 Measurement of absorbance

The absorbance of samples was measured using a spectrophotometer method according to Yang et al. (2020). Briefly, the samples were diluted 20‐fold with 0.1% (w/v) sodium dodecyl sulfate (SDS), then the absorbance of solutions was read at 294 nm and 420 nm, implying the absorbance of products generated at the intermediate and final stages of the Maillard reactions, respectively, using an ultraviolet (UV) spectrophotometer (SP‐UV 500DB spectrophotometer, Spectrum Instruments, Canada).

### 2.6 Degree of grafting (DG)

The degree of grafting was measured based on the TNBS method with some modifications (Qu et al., 2018). The solutions of produced conjugates (MLPH and MLPHC) were prepared at 0.2% (w/v) using 0.1% (w/v) SDS solution. Then, 0.4 mL of solutions was reacted with 2 mL of phosphate buffer (pH 8.2) and 1 mL of TNBS at 0.1% (w/v) for 30 min at 50°C in the dark. Thereafter, the reaction was terminated by the addition of 2 mL of 0.1 mol/L hydrochloric acid (HCl). After standing for 30 min, the absorbance of solutions was read at 340 nm using a UV spectrophotometer. DG was calculated as follows:

(1) $$\mathrm{DG}\;\left(\%\right)=\frac{\left(C_{0}-C_{t}\right)}{C_{0}}\times 100$$

where C_0_ (mmol/L) indicates the amino concentration of the ungrafted sample and C_t_ (mmol/L) is the amino concentration of the grafted sample at a grafting time *t* (h).

### 2.7 Surface hydrophobicity

Surface hydrophobicity (H_0_) of LPI, LPH, LPHC, MLPH, and MLPHC was determined using fluorescence intensity changes according to the method of Rezvankhah et al. (2021b) with a slight modification. On this basis, 1‐anilino‐8‐naphthalene sulfonate (ANS) was used as the hydrophobic fluorescence dye. Briefly, ANS dispersion was prepared at 8.0 mM in phosphate buffer and pH of 7.0. Then, 20 μL of the ANS dispersion was mixed with 2 mL of diluted sample solutions containing 0.01–0.1 mg/mL glycopeptides and kept for 2 min. In the next stage, the fluorescence intensity of the obtained mixtures was measured using a SpectraMax M3 spectrophotometer set at 390 and 470 nm as excitation and emission wavelengths, respectively. The slope of the net fluorescence intensity of glycopeptides with and without ANS versus glycopeptide content plot was determined and used as an index of H_0_.

### 2.8 Molecular weight (MW) profile

The molecular weight (MW) profiles of LPI, LPH, LPHC, MLPH, and MLPHC were analzyed according to the method of Rezvankhah et al. (2021a). SDS‐PAGE (polyacrylamide gel electrophoresis) was used to obtain the MW profile. Briefly, Laemmli sample buffer (Bio‐Rad) was used to dilute the samples. Then, 2‐mercaptoethanol (2‐ME) was incorporated into the sample buffer to provide a reducing condition. The obtained samples underwent heating for 4 min at 90°C. In the next stage, the samples were cooled and subjected to the electrophoresis at a constant voltage of 150 V by loading into 12% Mini‐PROTEAN® TGX™ Precast Gels (Bio‐Rad). Furthermore, MW standards (Bio‐Rad Broad Range Protein Molecular Weight Markers) were run alongside the samples as markers. An aqueous mixture of 40% (v/v) methanol, 10% (v/v) acetic acid, and 50% (v/v) deionized water containing 100 mg/mL Coomassie Brilliant Blue‐R‐250 was used to stain the gels for 2 h. Subsequently, an aqueous mixture of water (50%), methanol (40%), and acetic acid (10%) was used to discolor the gels.

### 2.9 Antioxidant activity

Antioxidant activity of produced samples was assessed according to the 2,2‐diphenyl‐1‐picrylhydrazyl (DPPH) assay (Rezvankhah, Yarmand, & Ghanbarzadeh, 2022). Briefly, an ethanolic DPPH solution (0.2 mM) was prepared and mixed with samples at 1 mg/mL. Then, the mixtures were stored at a dark place and room temperature (23°C) for 30 min. Subsequently, the prepared mixtures were analyzed for measuring the absorbance at 517 nm using a ultraviolet–visible (UV–vis) spectrophotometer. Also, a positive control such as ascorbic acid (0.01 mg/mL) was used to be compared with samples. The radical scavenging activity (RSA) was measured using the equation below:

(2) $$\text{Radical scavenging activity}\;\left(\mathrm{RSA},\%\right)=\frac{A_{C}-A_{S}}{A_{C}-A_{B}}\times 100$$

where *A*_C_, *A*_S_, and *A*_B_ indicate the absorbance values of control, sample, and blank, respectively.

### 2.10 Fourier transform infrared spectroscopy (FTIR)

The Fourier transform infrared (FTIR) spectra of lentil peptides conjugated with GA were recorded with a FTIR spectrophotometer (Perkin Elmer) from 400 to 4000 cm^−1^ at a resolution of 4 cm^−1^ (Rezvankhah et al., 2021a).

### 2.11 Volatile compounds

The head‐space solid‐phase microextraction (HS‐SPME) was used to purify the volatile compounds of samples (CTC Analytics, Zwingen, Switzerland). The separation was conducted using an Agilent 7890B gas chromatograph, while the identification was performed using an Agilent 5977A mass spectrometer (gas chromatography–mass spectrometry (GC–MS)) on the basis of the National Institute of Standards and Technology (NIST) database (Zha, Yang, et al., 2019). Initially, 1 g of samples was dissolved in 2 mL of distilled water. Then, the prepared solution was transferred into 20 mL capped glass vials, followed by preheating at 60°C for 10 min, using an autosampler heating block. A fiber needle with diameter of 50/30 μm (specified for SPME) was used for the injection. The prepared samples were injected into vial absorbing volatiles for 50 min. After that, the samples were transferred into the injector port (250°C, at spitless mode) for 3 min. A ZB‐Wax column (60 m × 0.25 mm × 0.25 μm, Agilent) was applied for GC–MS analysis. The SPME system was inserted into the injector while the temperature program was as follows: the oven temperature raised at 45°C/min from 40 to 85°C, then at 9°C/min from 85 to 200°C, and at 45°C/min from 200 to 250°C and held for 3 min isothermally. Helium as the carrier gas with ultra‐high purity was used at the flow rate of 1.5 mL/min. The electron impact mode (EI) was used for mass spectra, generating at 70 eV, while the scan range was from m/z 28 to 350. The volatiles were identified according to the mass spectra libraries.

### 2.12 Functional properties

#### 2.12.1 Emulsifying activity and stability indexes

Emulsifying activity and stability indexes (EAI and ESI) were ascertained according to the method of Mirzaee et al. (2023). Briefly, sample solutions were prepared at 10 mg/mL in glass tubes. Then, 10 mL of sunflower oil was added into 10 mL of solutions, followed by homogenization at 19000 rpm for 1 min using an Ultra‐Turrax (IKA T25). Subsequently, EAI was determined instantly after emulsion fabrication through 50 μL sampling from bottom of the emulsions. ESI was also ascertained after 10 min through 50 μL sampling from the bottom of the emulsions. Then, 5 mL of SDS (0.1%) was added into the taken volumes, followed by determining the absorbance of the diluted solutions at 500 nm using a spectrophotometer. Finally, EAI and ESI were ascertained using the equations as follows:

(3) $$\mathrm{EAI}\;\left(\frac{m^{2}}{g}\right)=\frac{\left(2\right)\left(2.303\right)\left(A_{0}\right)\left(\mathrm{DF}\right)}{\left(I\right)\left(\theta \right)\left(C\right)}$$

(4) $$\left(\mathrm{ESI}\right)\left(\min\right)=\frac{A_{0}}{∆A}∆t$$

where *A*_0_ exhibits the absorbance of the diluted emulsions instantly after homogenization, “DF” is the dilution factor (100), “I” is the path of length of cuvette (m), θ is the oil volume fraction (0.25), and “C” is the protein concentration in aqueous phase (g/m^3^); $∆A=A_{0}-A_{10}$, ∆t=10min.

#### 2.12.2 Foaming capacity and stability

Foaming capacity (FC) and foaming stability (FS) were determined according to the method of Mirzaee et al. (2023) with some modifications. Samples at 10 mg/mL were whipped at 19000 rpm for 2 min using an Ultra‐Turrax (IKA T25, Staufen, Germany). The aerated solutions were transferred into a 50 mL graded cylinder while measuring the total volume. The whipped solutions were also stored for 60 min at the ambient temperature and then the total volume was recorded. FC and FS were calculated using the equations below:

(5) $$\mathrm{FC}\;\left(\%\right)=\frac{B-A}{A}\times 100$$

(6) $$\mathrm{FS}\;\left(\%\right)=\frac{C-A}{A}\times 100$$

where A exhibits the volume of protein, peptide, and glycopeptide solutions before whipping (mL), B implies the instant volume of whipped solutions (mL), and C implies the volume of whipped solutions after storage of 60 min.

### 2.13 Sensory analysis

The sensory test was conducted for evaluating tastes including sweetness, saltiness, bitterness, and umami according to the procedure established by Rezvankhah et al. (2023). Briefly, six semi‐taught flavor specialists were employed for the taste evaluation and scored the samples from 1 to 7 based on the 7‐point hedonic method. The enriched umami soup samples were prepared by dissolving about 1% w/v of LPH, LPHC, MLPH, and MLPHC, followed by the addition of 0.5% w/v salt and 1% w/v MSG as the flavor enhancer. Besides, a mixture of LPI and umami soup (without produced samples) was considered as a reference to compare with the samples.

### 2.14 Statistical analysis

The experiments were carried out at three replications and data were reported as mean and SD. The statistical analysis of data was conducted by one‐way analysis of variance (ANOVA) and the mean difference analysis was performed by the Duncan test at the confidence level of 95% (*p* < .05).

## 3 RESULTS AND DISCUSSION

### 3.1 The absorbance of Maillard reaction products

**FIGURE 1:**
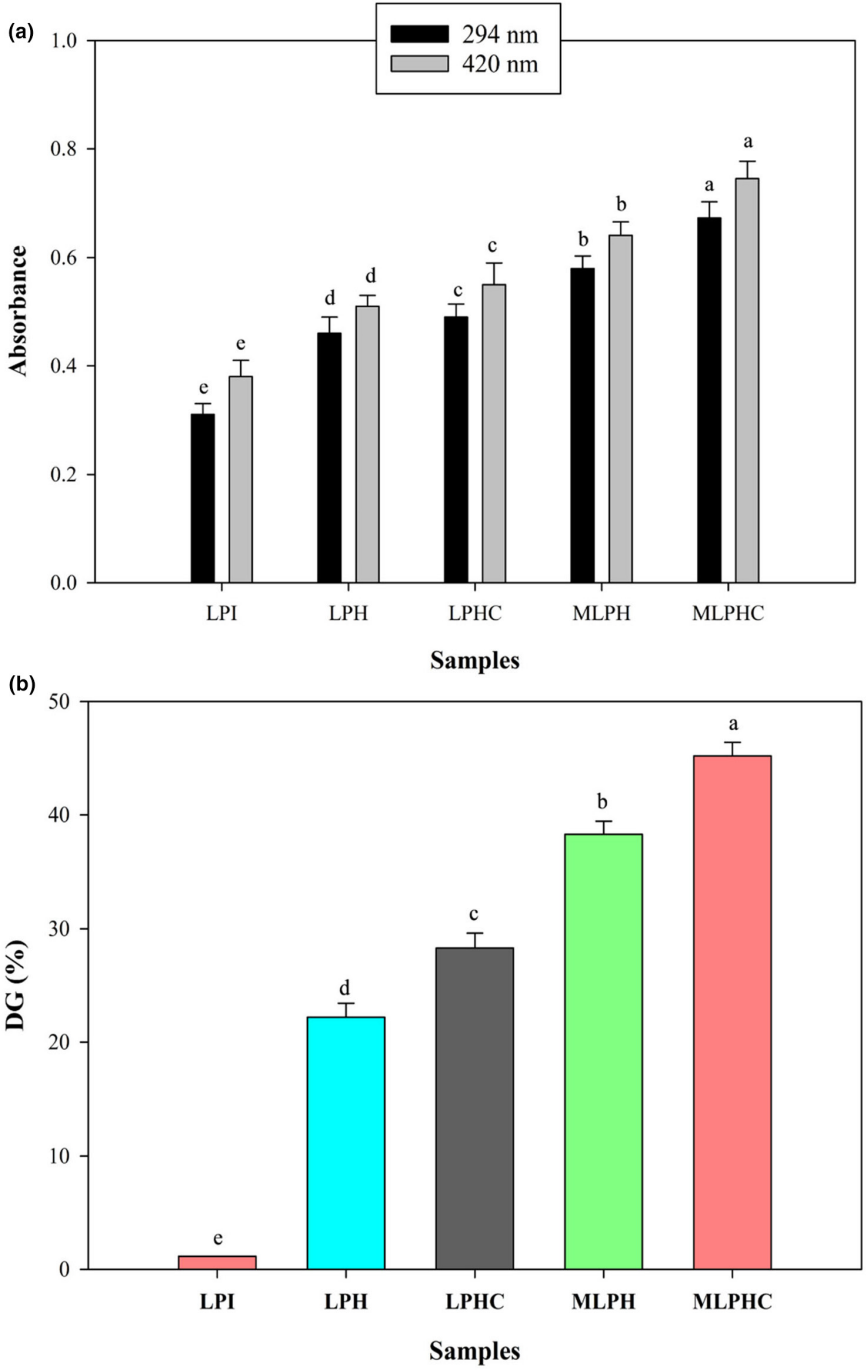
Absorbance at 294 nm and 420 nm at intermediate and final stages of the Maillard reaction (a) and degree of grafting (b) of LPI, LPH, LPHC, and MLPH, and MLPHC. The effects of hydrolysis of LPI, cross‐linking of LPH, and combined cross‐linking and Maillard reaction between LPH and LPHC with gum Arabic were investigated.

The absorbance of Maillard reaction products (MRPs) was investigated at two wavelengths (294 and 420 nm), which are implying the intermediate and final stages of the Maillard reactions, respectively. As is shown in Figure 1a, during the hydrolysis (LPH), cross‐linking, and Maillard reactions, the absorbance values of reaction products were significantly enhanced compared to control (LPI). In terms of LPH, the hydrolysis was conducted using Alcalase and Flavourzyme at 60 and 50°C and pH of 8.0 and 7.0, respectively. These circumstances individually induced the glycation between the free amino groups of the side chain of peptide molecules and carbonyl group of a reducing sugar (inherently bound to protein) or amino acids with free carbonyl groups, followed by browning reaction. The degree of browning can be an indicator of Maillard reaction's progress. The pH of 7.0 and 8.0 promoted the Schiff‐base formation, further increasing the browning rate during the hydrolysis reaction. During the hydrolysis, pH was dropped to lower extent, which decreased the intensity of browning. Thus, the hydrolysis can lead to Maillard reaction, even at moderate form. On another side, cross‐linking by MTGase (LPHC) led to a slight increase in the absorbance value of peptides (LPH) likely due to the polymerization that occurred between the peptide chains. Cross‐linking strengthened the protein structure and the pH drop rarely occurred and Maillard reaction was further promoted. When LPH was glycated with GA, the intensity of browning either in intermediate or final stages was increased and MLPH and, especially MLPHC, indicated the highest absorbance values. When the hydrolyzed LPI was glycated with GA, due to the presence of free amino groups, the rate of Maillard reaction was accelerated. Yang et al. (2020) reported that enzymatic hydrolysis produced peptides and amino acids in protein hydrolysate, easily involved in the Maillard reaction. The sugar cleavage produced colorless, small molecule mixtures, such as ketones, and aldehydes, which are capable of absorbing the ultraviolet–visible (UV–vis) light at 294 nm (Yang et al., 2020). The nitrogen‐containing brown polymers such as melanoidin directly contribute to the browning. The final or advanced stage related MRPs such as melanoidin can be determined by the reflection of color change of the reaction system at 420 nm. Simultaneously, when GA was used in conjunction with peptides (LPH and LPHC), the caramelization occurred, which deepened the color of the system. In some state, the increase in color intensity has been reported to decrease during the heating due to the degradation of peptide chains and the amino acid residues with color grafted with glucose, partly fading the color of the system. However, the color continued to deepen as the heating time increased, likely due to the degradation of the grafting product‐Amadori (Xiao et al., 2021).

### 3.2 Degree of grafting

Figure 1b illustrates the DG of LPH‐GA conjugates through wet‐heating. As shown, the sequential hydrolysis of LPI led to significant increase in DG (*p* < .05). It could be related to the conjugation of produced peptides and amino acids with free amino groups with free carbonyl groups of bounded sugar or amino acids with free carbonyl groups. MTGase‐mediated cross‐linking also increased the DG for LPHC, which could be related to grafting effects of MTGase and Maillard reaction. There are many reports that have declared hydrolysates contribute to Maillard reaction more potentially than native proteins. It is associated with presence of free amino groups that can interact with reducing sugars (Hu et al., 2023). Grafting or conjugation is not limited to Maillard reaction and MTGase can also form cross‐links between the peptide chains and increase the DG (Jiang et al., 2016; Wakabayashi et al., 2017). When LPH and LPHC were conjugated with GA, due to the presence of more reducing sites, the increase in DG was more promoted and reached the highest values for MLPH and MLPHC.

### 3.3 Surface hydrophobicity

**FIGURE 2:**
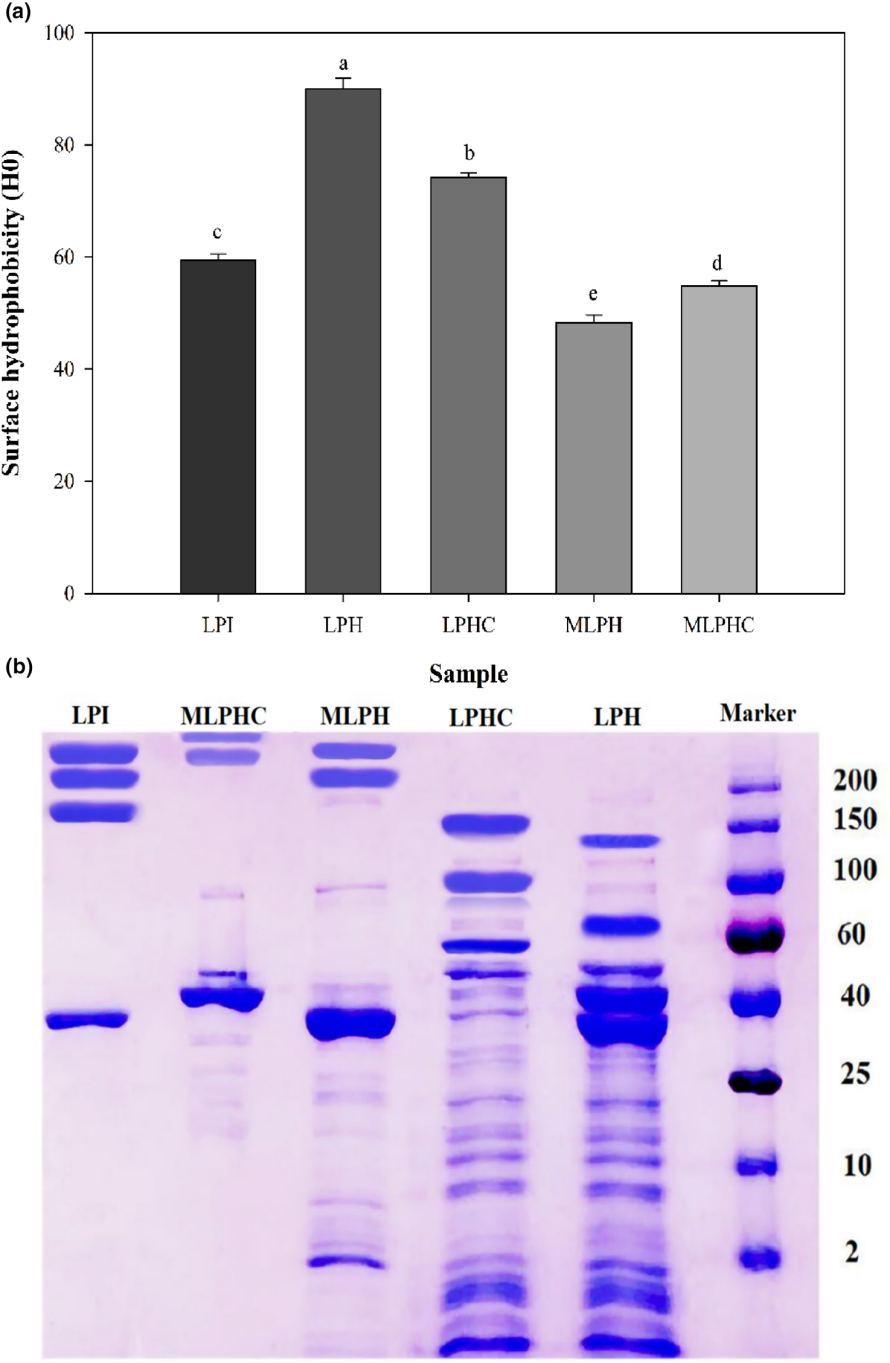
Surface hydrophobicity by fluorescence dye of ANS (a) and MW profiles by SDS‐PAGE (b) of LPI, LPH, LPHC, MLPH, and MLPHC. The effects of hydrolysis of LPI, cross‐linking of LPH, and combined cross‐linking and Maillard reaction between LPH and LPHC with gum Arabic were investigated.

Surface hydrophobicity of proteins and their derived peptides remarkably influences the emulsification and also sensory properties (Liu, Pei, & Heinonen, 2022; Mirzaee et al., 2023; Rezvankhah, Yarmand, & Ghanbarzadeh, 2022). The proteins and peptides have amphiphilic nature, which allows them to remain in the aqueous phase while simultaneously adsorbing at the interface and generate stabilizing electrostatic forces and steric hindrance (Emam‐Djomeh & Rezvankhah, 2020; Jafari, 2020). The hydrophobic patches of proteins and peptides, which are associated with the hydrophobic amino acid residues, support the protein spatial displacement at the oil–water interface (Liu, Pei, & Heinonen, 2022; Rezvankhah et al., 2023). The results of the surface hydrophobicity are presented in Figure 2a. The order of the surface hydrophobicity values was given as LPH > LPHC > LPI > MLPHC > MLPH. The main reason for high H_0_ of LPH might be attributed to exposing more buried hydrophobic segments after hydrolysis by Alcalase–Flavourzyme. Rezvankhah et al. (2021b) reported that the hydrolysis of lentil protein can unravel the spatial structure of protein and support exposing the buried hydrophobic groups. Lentil protein is rich in hydrophobic amino acids, such as valine, alanine, leucine, isoleucine, proline, methionine, cysteine, and glycine (Rezvankhah et al., 2021a, 2021b). The protein structure has inherently buried these amino acids. Thermal treatment or more precisely denaturation (holding at 85–95°C for 15–30 min) and hydrolysis of proteins alter their spatial conformations and force the hydrophobic segments to locate at the surface (Liu, Pei, & Heinonen, 2022). Hence, heat‐treated LPI showed relatively higher H_0_ than non‐treated LPI (not shown). Although LPHC indicated higher H_0_ than LPI, MLPH, and MLPHC, MTGase‐mediated cross‐linking led to reburying the exposed hydrophobic segments, and reducing the surface hydrophobicity. It was in line with results found in our previous research (Rezvankhah et al., 2023; Rezvankhah, Yarmand, & Ghanbarzadeh, 2022). Cross‐linking by MTGase can rebury the hydrophobic sites exposed previously by heat treatment and enzymatic hydrolysis (Zheng et al., 2015). On the other side, conjugation of LPH and LPHC with GA led to significant reduction of H_0_, which could be attributed to inherent hydrophilic nature of GA. Indeed, when GA with predominant hydrophilic nature is linked to proteins or peptides, the l‐arabinose, l‐rhamnose, and d‐glucuronic acid and 1,3‐linked β‐d‐galactopyranosyl units coat the hydrophobic patches of proteins/peptides, thus reducing surface hydrophobicity (Qu et al., 2018; Xiao et al., 2021; Yang et al., 2020; Zha, Yang, et al., 2019). Zha, Dong, et al. (2019) reported that the grafting of GA to pea protein concentrate increased the surface hydrophilicity and steric hindrance of glycoprotein. Similarly, when pea protein hydrolysates were cross‐linked with GA through the Maillard reaction, surface hydrophobicity was decreased (Zha, Yang, et al., 2019).

### 3.4 MW profile

The MW profiles of unhydrolyzed LPI, LPH, LPHC, MLPH, and MLPHC are presented in Figure 2b. One intense band was observed for LPI at MW close to 35 kDa and three other bands were formed at 150, 200, and >200 kDa. The first band was assigned to the acidic subunit of 11S (legumin‐like) (Jarpa‐Parra et al., 2014; Rezvankhah et al., 2021b; Zha, Yang, et al., 2019). Barbana and Boye (2011) reported that the peptide bands detected at >85 kDa were ascribed to legumin and vicilin fractions. In terms of the MW profile of LPH, sequential hydrolysis of LPI formed peptides with <2 kDa, 2–10 kDa, 10–25 kDa, 25–40 kDa (two sharp bands at 33 kDa and 40 kDa), 40–60 kDa, and 100–150 kDa. The peptides with MW of 2–10 kDa could be related to 2S albumin that constitutes a light (~4.5 kDa) and a heavy (~10 kDa) polypeptide chain (Zha, Yang, et al., 2019). The peptides with MW around 10–15 kDa could be related to albumin polypeptides, γ‐vicilin, and respective derived fractions (Rezvankhah et al., 2021b, 2023; Zha, Dong, et al., 2019). The peptides at 15–25 kDa could be related to subunits of 7S vicilin or gamma‐vicilin storage proteins (Rezvankhah et al., 2021a; Rezvankhah, Yarmand, & Ghanbarzadeh, 2022; Zha, Dong, et al., 2019). The major bands that electrophoretically indicated were at 30–35 kDa, 35–40 kDa, and 60 kDa, ascribed to the subunits of vicilin (48 kDa) and convicilin (63 kDa) (Rezvankhah et al., 2021a; Rezvankhah, Yarmand, & Ghanbarzadeh, 2022; Zha, Dong, et al., 2019). In the case of LPHC, MTGase‐mediated cross‐linking formed new peptides with enlarged molecular size. Indeed, the bands detected for LPH were shifted to larger MWs. As shown in Figure 2b, the former bands at 35–40 kDa and 60 kDa were shifted to 50 and close to 100 kDa while the band at 100–150 approached 150 kDa. This was in line with other research reports that MTGase‐mediated cross‐linking promoted the polymerization and larger peptides were generated (Rezvankhah, Yarmand, & Ghanbarzadeh, 2022; Wang et al., 2016; Zhang, Chen, et al., 2021; Zhang, Cheng, et al., 2021). To assess the heat treatment (Maillard reaction) effects on the MW profile of combination of hydrolysates with GA, LPH and LPHC were conjugated with GA through the Maillard reaction and MLPH and MLPHC were developed. For MLPH, a sharp band was detected at around 35 kDa and two sharp bands were detected at 200 kDa and >200 kDa, confirming the conjugation of LPH and GA. Also, most of the sharp bands at <2 kDa, 2–10 kDa, 10–25 kDa, 40 kDa, 40–60 kDa, and 100–150 kDa of LPH had disappeared or the respective intensities were decreased while two bands were formed at higher MWs for MLPH. In this regard, Zha, Dong, et al. (2019) applied the controllable Maillard reaction to modify the pea protein concentrate with GA. They reported that the newly formed band near the loading end of gel concomitant with disappearance of some pea protein subunits was observed. Moreover, large MW biopolymers are not capable of migrating into the separating gel, possibly due to MW of GA (>250 kDa) that was conjugated with LPH, and formed a higher MW conjugate. Hence, the electrophoretic profile indicated newly formed glycopeptides. On another side, Maillard‐mediated cross‐linking of LPHC and GA led to higher MWs than MLPH. According to the electrophoretic profile of MLPHC, a sharp band was observed at around 40 kDa and also two bands with higher MWs than MLPH were detected, ascribed to the higher MW of LPHC than LPH. As a consequence, conjugation of LPHC with GA led to the development of MLPHC with higher MWs than MLPH, which was in line with Zha, Dong, et al. (2019). The initial cross‐linking using MTGase promoted the polymerization while when LPHC was conjugated with GA, the MW was significantly increased and the band was shifted to MW > 250 kDa.

### 3.5 DPPH radical scavenging activity

**FIGURE 3:**
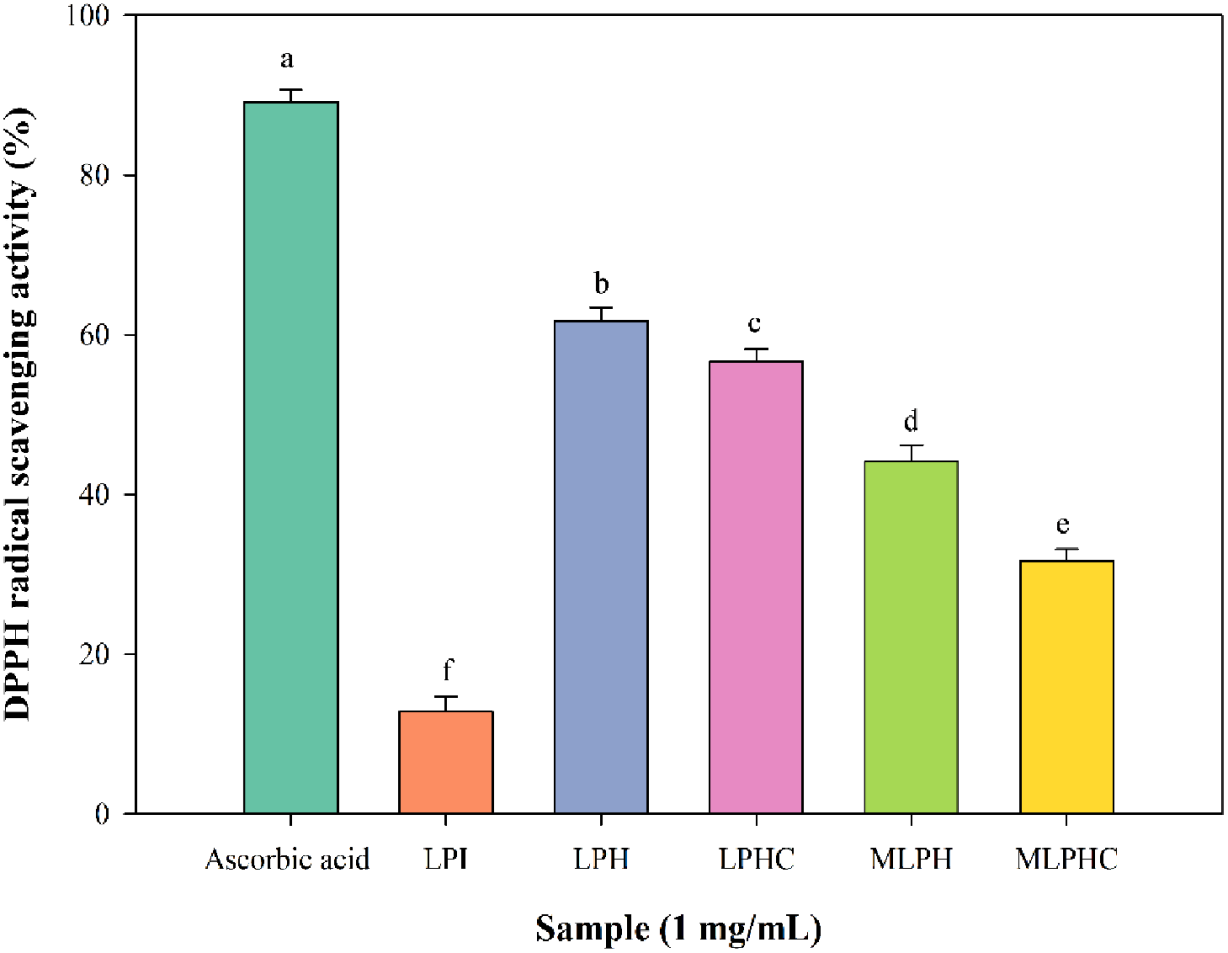
Antioxidant activity by DPPH method for LPI, LPH, LPHC, MLPH, and MLPHC. The effects of hydrolysis of LPI, cross‐linking of LPH, and combined cross‐linking and Maillard reaction between LPH and LPHC with gum Arabic were investigated.

Antioxidant activity of hydrolysates and conjugated hydrolysates was determined and the results are illustrated in Figure 3. LPI indicated DPPH RSA of 12.77%, while hydrolysis significantly increased the antioxidant activity and reached 61.77% (*p* < 0.05). The antioxidant activity of LPI could be related to antioxidant amino acids that are abundantly and intrinsically present (Avramenko et al., 2013; Rezvankhah et al., 2021b). Lysine, leucine, isoleucine, tyrosine, phenylalanine, histidine, glutamic and aspartic acids have been reported as predominant amino acids of LPI (Rezvankhah et al., 2021a). Some of these amino acids are hydrophobic (leucine, isoleucine, histidine, and phenylalanine) which can potentially interact with hydrophobic DPPH radicals and some of them are hydrophilic while negatively charged (glutamic and aspartic acids) which can have electron‐donating potential (Mirzaee et al., 2023; Rezvankhah, Yarmand, & Ghanbarzadeh, 2022). However, most of the antioxidant amino acids are buried in intact form of LPI (Parolia et al., 2022). When LPI is thermally heated, the hydrophobic segments are exposed to the surface of protein and the interaction of antioxidant parts is increased with free reactive radicals (Avramenko et al., 2013; Mirzaee et al., 2023; Rezvankhah et al., 2021b). In addition, when LPI is hydrolyzed by proteolytic enzymes, more potent bioactive peptides are produced, which have lower MW and high antioxidant amino acid residues (Avramenko et al., 2013; Rezvankhah et al., 2023). More specifically, sequential hydrolysis has shown impressive effects on the biological activities of produced peptides (Ozón et al., 2022; Rezvankhah et al., 2023; Xu et al., 2020). Alcalase and Flavourzyme have been known as the most industrial enzymes in the production of bioactive peptides from vegetable proteins, aiming at production of umami taste development (Rezvankhah et al., 2023; Wei et al., 2018). MTGase‐mediated cross‐linking of LPH that produced newly formed peptides (LPHC) significantly decreased the antioxidant activity (56.63%) (*p* < .05) (Figure 3). MTGase reburied the unraveled structure of peptides, thus decreasing the interaction of hydrophobic patches with DPPH radicals (Rezvankhah et al., 2023; Rezvankhah, Yarmand, & Ghanbarzadeh, 2022). Similar results were reported for the cross‐linking of soybean protein hydrolysates (Zhang, Chen, et al., 2021; Zhang, Cheng, et al., 2021). They reported that post‐hydrolysis cross‐linking rearranged the (poly)peptides in soy protein hydrolysates via glutaminyl modifications, followed by reduction of surface hydrophobicity. He et al. (2021) partially hydrolyzed the zein by trypsin and then conjugated by MTGase to chitosan oligosaccharide lactate. Accordingly, the hydrolysis and subsequently, conjugation decreased the surface hydrophobicity by more than 20% (He et al., 2021). The surface hydrophobicity is an important factor in increasing DPPH RSA, which is related to the inherent hydrophobic properties of DPPH radicals. Maillard conjugation led to significant reduction of DPPH RSA while it reached 44.14% and 31.66% for MLPH and MLPHC, respectively. There are diverse findings regarding the Maillard effects on the antioxidant activity, especially in terms of the conjugation of peptides and carbohydrates (Fadel et al., 2023; Liu et al., 2012; Yu et al., 2018). The volatile compounds and low MW heterocyclic compounds might be attributed to the increase of antioxidant activity after Maillard reaction between soybean protein hydrolysates and xylose (Yu et al., 2018). In addition, peptides with 3–5 kDa were easy to cross‐link with xylose to develop polymers with high antioxidant activity. High MW melanoidins and low MW heterocyclic compounds generated from Maillard reaction could be mainly responsible for the antioxidant activity (Yu et al., 2018). However, our results were in contrast to findings of previous studies. It was discovered that not only the MRPs have substantial impacts on antioxidant activity, but surface hydrophobicity of final products should also be taken into account. Cross‐linking of GA with LPH and LPHC presumably coated the exposed hydrophobic segments of hydrolysates, thus, reduction of surface hydrophobicity and reduction of interactions with DPPH radicals. Participation of some of the antioxidant amino acid residues in Maillard reaction has demonstrated reduction of antioxidant activity (Fadel et al., 2023; Zha, Yang, et al., 2019). Therefore, grafting GA with LPH and LPHC formed a strong steric hindrance that decreased the surface hydrophobicity, which was in line with findings of previous studies (Dai et al., 2023; Xia et al., 2023). Lysine and arginine are antioxidant amino acids with first amine that participates in the Maillard reaction which can serve as the cause of antioxidant activity reduction. When the antioxidant amino acid residues are located at C‐terminal or N‐terminal, the interaction of peptides with DPPH radicals is increased while by Maillard reaction, these amino acids are run out of available stock due to grafting with anomeric carbon of sugars (Mu et al., 2010). Also, when peptides are grafted with GA, due to the alteration of third structure (hydrophobic interactions), the aromatic amino acids are also altered, followed by reduction in antioxidant activity (Pirestani et al., 2018). It should also be noted that the reduction of DPPH RSA might be related to inherent hydrophobic properties of DPPH radicals. When the hydrophobic sites are covered, the interaction with DPPH radicals is decreased (Rezvankhah et al., 2023; Rezvankhah, Yarmand, & Ghanbarzadeh, 2022). Therefore, the conjugated hydrolysates exhibited lower antioxidant activity compared to ascorbic acid.

### 3.6 FTIR analysis

**FIGURE 4:**
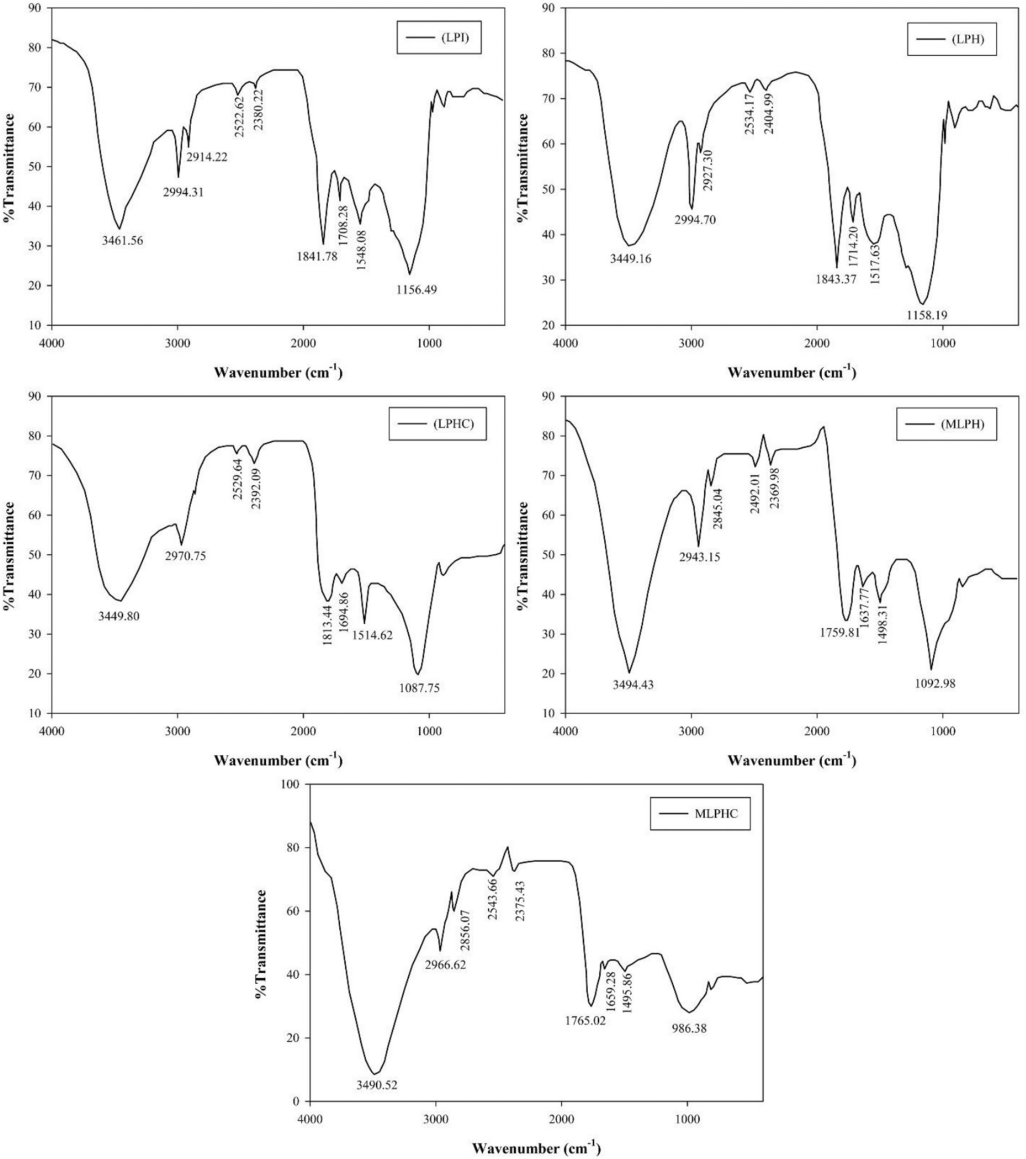
Functional groups by FTIR for LPI, LPH, LPHC, MLPH, and MLPHC. The effects of hydrolysis, cross‐linking, and combined cross‐linking and Maillard reaction were investigated.

The functional groups of hydrolysates and grafted hydrolysates were determined by FTIR and spectrums are represented in Figure 4. Regarding the chemical spectrum of LPI, a peak was detected at 3461 cm^−1^, which can be attributed to first amine (‐NH_2_) or hydroxyl (‐OH) groups. A stretching band was observed at 2994 cm^−1^, which can be attributed to methyl (‐CH_3_) and methylene groups (‐CH_2_) that are present in the structure of peptides (Rezvankhah, Emam‐Djomeh, et al., 2022). A stretching peak was also detected at 1841 cm^−1^ followed by a short peak at 1708 cm^−1^ which are related to carbonyl groups (C=O), originated from the amide bonds (CO‐NH_2_) or carboxylic groups (COOH) (Rezvankhah, Emam‐Djomeh, et al., 2022; Tian et al., 2020). The frequency of the first amide band is so sensitive to secondary structure of protein such as α‐helix, β‐turn, β‐sheet, and random coil (Tian et al., 2020). Hence, the first amide band is considered as the fingerprint for secondary structure of proteins (Tian et al., 2020). The spectral frequency was detected at 1548 cm^−1^, which is attributed to second amide band vibration originating from C‐N and N‐H deformation (Tian et al., 2020; Zha, Yang, et al., 2019). The spectral band was observed at 1156 cm^−1^, which may result from the out‐of‐plane C‐H bending vibration, originating from the third structure of proteins and likely from β‐sheet (Zha, Yang, et al., 2019). According to Xie et al. (2019), the presence of amide bands can demonstrate the bonds of C=C, C=N, ‐COOH, and ‐OH.

Sequential hydrolysis slightly shifted the bands while the intensity of bands was also altered. According to the FTIR spectra of LPH, the observed stretching band at 3449 cm^−1^ was broader than that observed for LPI, which may result from the exposure of more ‐NH_2_ or ‐COOH groups after hydrolysis. The spectral band detected at 2994 cm^−1^ was more stretched, which could be attributed to the exposure of buried hydrophobic patches (‐CH_2_‐CH_2_‐). A stretching band was observed at 1843 cm^−1^, which may result from carbonyl vibration related to amide I structure (CO‐NH_2_). The peak at 1708 cm^−1^ was shifted to 1714 cm^−1^ while the intensity of peak was slightly increased, attributed to the secondary structure alteration. The spectral band at 1517 cm^−1^ was shifted from 1548 cm^−1^, related to the bending vibration of C‐N and N‐H of second amide (Mattice & Marangoni, 2021). These alterations showed that the secondary structure of LPI was changed after hydrolysis. A stretching band was observed at 1158 cm^−1^, showed a slight shift, and related to amide III (Mattice & Marangoni, 2021).

According to FTIR spectra of LPHC (Figure 4), the intensity of broad peak at 3449 cm^−1^ was decreased, which was attributed to the reburying of unraveled groups (‐OH and ‐NH_2_). When LPH underwent the cross‐linking with MTGase, the lysine and glutamine amino acid residues were covalently bonded, and the exposed segments were buried (Rezvankhah, Yarmand, & Ghanbarzadeh, 2022; Wang et al., 2016; Zhang, Chen, et al., 2021; Zhang, Cheng, et al., 2021). The spectral band at 2970 cm^−1^ was attributed to the hydrophobic ‐CH_2_ and CH_3_ while the peak indicated a slight shift and the frequency of peak was decreased. It could be related to the MTGase‐catalyzing action that caused reburying and consequently, the exposed hydrophobic patches were covered (Rezvankhah et al., 2023; Rezvankhah, Yarmand, & Ghanbarzadeh, 2022). The spectral bands at 1813 and 1694 cm^−1^ indicated shifts while also having lower intensity compared to that obtained for LPH. In contrast, the peak detected at 1514 cm^−1^ exhibited more stretching state than that observed for LPH. These alterations displayed the changes occurred for amide I and amide II structures (Mattice & Marangoni, 2021). Zhang, Chen, et al., 2021; Zhang, Cheng, et al., 2021 investigated the combining of the hydrolysis of soybean protein by Alcalase and cross‐linking of hydrolysates by transglutaminase and reported that MTGase‐catalyzing of peptides led to increase of N‐H bending vibration originated from isopeptide formation. Zhang, Chen, et al., 2021; Zhang, Cheng, et al., 2021 reported that cross‐linked peanut protein by transglutaminase led to some changes in the second structure as follows: β‐sheet > α‐helix > β‐turn > random coil. According to Figure 4, a sharp stretching peak was detected at 1087 cm^−1^ that was shifted from 1158 cm^−1^ and illuminated the third structure of proteins which was altered as confirmed by the surface hydrophobicity value. This peak is attributed to the out‐of‐plane C‐H bending vibration and the reburying effect of MTGase led to a reduction of respective intensities (Zha, Yang, et al., 2019).

In terms of FTIR spectra of MLPH with respect to the conjugated LPH and GA (Figure 4), a sharp stretching peak was observed at 3494 cm^−1^, which may result from the hydroxyl and amine groups of GA and LPH, respectively. Grafting GA with LPH increased the number of ‐OH and thus, the frequency of peak was increased. Also, conjugation of LPH with GA also increased the noncovalent bonds such as hydrogen linkages; thus, the increase in frequency of the detected peak could be attributed to the increase in hydrogen bond formation (Pirestani et al., 2018; Rezvankhah et al., 2021a). A sharp peak was observed at 2943 cm^−1^ that was attributed to the methyl and methylene groups originated from asymmetric C‐H stretching (Rezvankhah, Emam‐Djomeh, et al., 2022). Conjugation of LPH with GA decreased the intensity of this peak, as confirmed by surface hydrophobicity (Dai et al., 2023; Yang et al., 2020). A spectral peak was detected at 1759 cm^−1^ with lowered intensity that also showed shifting from 1843 cm^−1^. It should also be noted that the peaks detected at 1700–1760 cm^−1^ can demonstrate the Maillard reaction occurring and likely the peak at 1843 cm^−1^ had disappeared after grafting (Chiang et al., 2022; Yang et al., 2020). The peaks including 1637 and 1498 cm^−1^ with lowered intensities were detected related to amide I and amide II. Pirestani et al. (2018) produced canola protein–GA conjugate and reported that the absorbances of amide I, II, and III bands of conjugate were decreased and shifted from wavenumbers 1645, 1538, and 1238 cm^−1^ in canola protein to 1648, 1546, and 1248 cm^−1^, respectively. The detected peak at 1158 cm^−1^ for LPH was shifted to 1092 cm^−1^ for MLPH. Reports have demonstrated that after glycation reaction with polysaccharides, the functional groups of proteins may be changed and new groups in MRPs including Amadori compound (C=O), Schiff base (C=N), and pyrazines (C‐N) may be formed (Pirestani et al., 2018).

According to the FTIR spectrum of MLPHC that was developed from grafting LPHC with GA (Figure 4), a broad stretching band was detected at 3490 cm^−1^ with higher intensity than that obtained for LPHC. By the grafting of LPHC with GA, the hydrogen bonds with peptides were increased. Also, a short peak was observed at 2966 cm^−1^ with similar intensity to that obtained for LPHC. A spectral band was observed at 1765 cm^−1^, which could indicate the Maillard reaction taking place. The intensity of peaks detected at 1659 and 1495 cm^−1^ was decreased, which demonstrated the changes in amides I and II of LPH after cross‐linking with MTGase, followed by grafting with GA (Pirestani et al., 2018; Zha, Yang, et al., 2019). Moreover, a broad peak was detected at 986 cm^−1^, which distinguished the MLPHC from other samples. This band likely arose from C‐H deformation, C‐O, and C‐C stretching found in GA (Pirestani et al., 2018).

### 3.7 Volatile components’ determination

**FIGURE 5:**
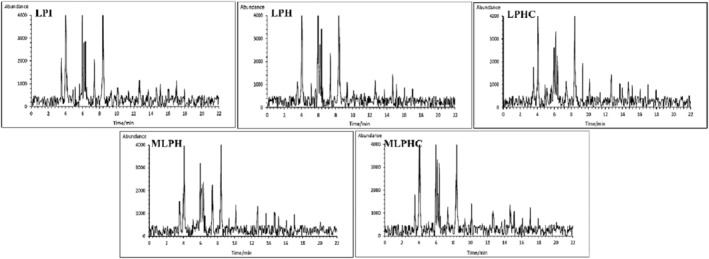
Volatile compounds detected chromatograms of LPI, LPH, LPHC, MLPH, and MLPHC by GC–MS. The effects of hydrolysis of LPI, cross‐linking of LPH, conjugation of LPH with gum Arabic, and combined cross‐linking and conjugation of LPH with LPH.

The identified and quantified volatile compounds in pulses include aromatic hydrocarbons, aldehydes, alkanes/alkenes, alcohols, ketones, acids, esters (without lactones), pyrazines, terpenes, furans, and lactones (Devaere et al., 2022; Karolkowski et al., 2021). In the present study, the effects of sequential hydrolysis of LPI by Alcalase and Flavourzyme, cross‐linking of LPH by MTGase, grafting through the Maillard reaction (MLPH), and combined cross‐linking and Maillard conjugation (MLPHC) were investigated. The alterations of volatile components were determined by GC–MS and quantified values based on their retention times (Figure 5) are presented in Table 1. Accordingly, 1‐Penten‐3‐ol was found as an alcohol, penetrating grassy ethereal odor, which was decreased after hydrolysis, cross‐linking, and Maillard conjugation. It is originated from fatty acids’ breakdown and further processing indicated decreasing impacts on the concentration. Hexanal, as an aldehyde, is originated from breakdown of fatty acids during the oxidation of pulses. Its odor describes grassy, floral, fruity, and fresh grass. Hexanal is mainly derived from 13‐hydroperoxide degradation. As shown in Table 1, Hexanal is one of the major volatiles and its concentration was increased after hydrolysis, likely due to the exposure of buried segments with high concentration of hexanal linked. MTGase‐mediated cross‐linking and Maillard grafting decreased the hexanal concentration while combined cross‐linking and Maillard conjugation increased Hexanal. A similar trend was observed for (E)‐2‐Hexanal, while the MLPHC indicated the lowest concentration. As another alcohol, 3‐Methyl‐1‐butanol, penetrating green aroma, comes from amino acid degradation. Cross‐linking by MTGase concentrated this volatile, while hydrolysis and Maillard reaction decreased it. A similar trend was observed for 1‐Pentanol, as one of the major alcohols identified in pulses, and is originally derived from the fatty acid breakdown (Karolkowski et al., 2021). 2‐Pentylfuran, classified as ketone, has green bean, metallic, vegetable, sweet rum odors with caramel‐like and cocoa notes, which is found in the protein concentrates. It was observed that the hydrolysis, cross‐linking, and Maillard conjugation increased the 2‐Pentylfuran concentration (Table 1). 1‐Octen‐3‐one was one of the major volatiles identified in the samples. Its concentration was decreased after hydrolysis, cross‐linking by MTGase, and conjugation with GA. 3‐Octen‐2‐one was increased after hydrolysis, decreased after cross‐linking, while it increased after combined MTGase‐mediated cross‐linking and Maillard conjugation. Pulse proteins, such as pea, chickpea, and lentil, have been reported to have eight times more ketones than dehulled peas or lentils, with a high presence of 2,3‐pentanedione, 5‐hexen‐2‐one, heptanone, 2‐methyl‐3‐heptanone, 3‐octen‐2‐one, and 2,3‐octanedione (Karolkowski et al., 2021). According to the reports, these volatiles are derived from the free fatty acids’ breakdown during the oxidation (Karolkowski et al., 2021; Rezvankhah et al., 2019). Their production is promoted by an increase in temperature during the wet process that accelerates the autoxidation (Karolkowski et al., 2021; Rezvankhah et al., 2018). The 1‐Hexanol, (Z)‐2‐Heptanol, 1‐Octen‐3‐ol, 1‐Heptanol, 1‐Octanol, (E)‐2‐Octen‐1‐ol, 1‐Nonanol, and (Z)‐3‐Nonen‐1‐ol were increased after MTGase‐mediated cross‐linking while hydrolysis and combined cross‐linking and Maillard conjugation indicated different impacts. Benzaldehyde, Benzeneacetaldehyde, (E,E)‐2,4‐Nonadienal, 10‐Undecenal, Tridecanal, and (E,E)‐2,4‐Decadienal were aldehydes that mainly originated from amino acids and fatty acid degradation. Hydrolysis, MTGase‐mediated cross‐linking, and combined cross‐linking and Maillard conjugation indicated different effects which could be attributed to the exposure after hydrolysis, reburying after MTGase treatment, and covering effects after Maillard reaction. Two organic acids including Butanoic and Hexanoic acids were identified, while MTGase‐mediated cross‐linking and combined cross‐linking and conjugation potentially influenced the concentration. Hydrolysis, cross‐linking, and Maillard conjugation indicated increasing effects while combined cross‐linking and Maillard reaction decreased the concentration of identified volatiles. Karolkowski et al. (2021) reported that dehulling of pulses exposes lipids or free fatty acids to more oxygen and promotes lipid degradation. Due to the presence of phenolic compounds in hulls, the oxidation is limited through the antioxidant activity. Lipid oxidation promotes the production of aldehydes, ketones, and alcohols, which influence the sensory properties of proteins. According to Table 1, hydrolysis, MTGase‐mediated cross‐linking, Maillard reaction, and combined MTGase‐mediated and Maillard reaction showed significant effects on the concentration of volatile compounds. These changes led to production of novel conjugates with improved sensory properties (reduction of grassy and beany tastes and development of umami taste). Indeed, volatile components can influence the tastes of peptides. When the unfavorable volatile components were changed by further processing, the peptides strongly contributed to the meaty‐analog flavor. On this basis, MLPH and MLPHC exhibited well‐balanced volatile components, which led to the development of umami taste which the complementary sensory analysis confirmed.

**TABLE 1:** Volatile compounds obtained by GC–MS for lentil protein isolate, hydrolysates, and hydrolysates cross‐linked by MTGase, conjugated with gum Arabic, and combined cross‐linking and conjugation of hydrolysates with gum Arabic.

Volatile compounds (%)	Retention time (min)	LPI	LPH	LPHC	MLPH	MLPHC
1‐Penten‐3‐ol	3.54	5.418	2.731	4.545	4.194	4.411
Hexanal	4.05	12.611	16.151	12.501	10.858	15.378
3‐Methyl‐1‐butanol	4.91	1.835	1.115	2.542	0.568	1.281
(E)‐2‐Hexanal	5.13	2.206	2.596	2.169	2.031	1.564
2‐Pentylfuran	5.52	0.529	0.847	1.511	1.522	1.408
1‐Pentanol	5.65	2.563	1.987	2.46	1.808	1.399
1‐Octen‐3‐one	5.98	12.276	10.726	6.704	8.776	10.338
1‐Hexanol	6.16	6.149	6.193	8.391	5.833	8.116
Acetoin	6.25	2.507	2.011	1.535	2.50	2.007
3‐Octen‐2‐one	6.36	7.143	8.225	5.769	6.467	7.623
(Z)‐2‐Heptanol	6.50	2.191	2.106	1.033	2.471	2.131
Nonanal	7.39	5.238	5.697	2.937	6.216	3.111
1‐Octen‐3‐ol	8.41	12.436	12.998	13.83	15.974	12.412
1‐Heptanol	9.36	1.814	2.657	4.999	2.218	1.837
1‐Octanol	10.13	2.113	1.806	3.204	3.752	3.469
3,5‐Octadien‐2‐one	10.51	1.001	1.368	1.192	0.621	0.994
Benzaldehyde	11.01	0.848	1.431	1.206	0.951	0.859
(E)‐2‐Octen‐1‐ol	11.39	1.762	1.516	1.666	1.372	1.297
1‐Nonanol	12.71	2.911	1.998	3.658	3.654	2.108
Benzeneacetaldehyde	13.10	1.253	1.238	0.551	1.282	0.726
Butanoic acid	13.71	1.869	1.898	2.672	2.80	1.524
(Z)‐3‐Nonen‐1‐ol	14.01	1.056	1.039	2.255	1.284	1.741
(E,E)‐2,4‐Nonadienal	14.68	2.045	3.384	2.877	2.869	3.325
10‐Undecenal	15.14	2.50	2.462	2.479	2.385	2.641
Hexanoic acid	16.11	1.92	2.109	2.082	1.885	1.905
Tridecanal	17.02	2.872	1.95	2.581	2.647	3.036
(E,E)‐2,4‐Decadienal	17.95	1.932	1.122	1.936	1.356	1.88
1‐Pentadecanol	20.09	1.003	0.54	0.711	1.706	1.48

### 3.8 Emulsifying properties

Emulsifying activity and stability indexes of LPI, hydrolyzed LPI, cross‐linked LPH, grafted LPH, and LPHC with GA were investigated at varying pH values (2.0, 4.0, 6.0, 8.0, and 10.0) and the results are represented in Table 2. The lowest EAI and ESI were achieved when pH was set at 4.0, as the isoelectric point of protein with the net charge of zero and electrostatic attraction is predominant. At isoelectric point, MLPH indicated the highest EAI and ESI, while LPH and LPHC exhibited the lowest values, likely due to the misbalancing of the hydrophobic/hydrophilic segments by hydrolysis and cross‐linking. Thus, conjugation illuminated how grafting of LPH and LPHC with GA can improve the emulsifying properties of hydrolysates. It is due to the fact that GA has been known as an efficient emulsifier and stabilizer that has negative charges in a vast range of pH (2–8) (Rezvankhah et al., 2020; Zha, Yang, et al., 2019). Therefore, complexation of LPH and LPHC with GA significantly improved EAI and ESI due to increase in steric hindrance and repulsive forces (Rezvankhah, Emam‐Djomeh, et al., 2022; Zha, Dong, et al., 2019).

**TABLE 2:** The effects of hydrolysis of LPI, cross‐linking of hydrolysates, hydrolysates conjugated with gum Arabic, and combined cross‐linking and Maillard conjugation of hydrolysates with GA on EAI (m^2^/g) and ESI (min).

Sample	Emulsifying activity (m^2^/g) at varying pH values				
	2.0	4.0	6.0	8.0	10.0
LPI	33.34 ± 0.30^Cb^	22.98 ± 0.23^Db^	34.22 ± 0.61^Cc^	40.18 ± 0.93^Bb^	66.29 ± 0.45^Ac^
LPH	33.15 ± 0.71^Cb^	20.20 ± 0.82^Ec^	31.69 ± 0.59^Dd^	34.95 ± 0.62^Bd^	53.83 ± 1.18^Ae^
LPHC	33.51 ± 0.81^Db^	19.78 ± 0.65^Ed^	36.50 ± 0.94^Cb^	38.65 ± 0.56^Bc^	57.53 ± 0.47^Ad^
MLPH	35.56 ± 0.13^Da^	27.55 ± 0.89^Ea^	41.96 ± 1.17^Ca^	46.41 ± 0.87^Ba^	73.76 ± 1.02^Aa^
MLPHC	31.67 ± 0.10^Dc^	23.96 ± 0.97^Eb^	37.43 ± 0.58^Cb^	41.06 ± 0.55^Bb^	70.12 ± 0.34^Ab^

	Emulsifying stability (min) at varying pH values				
	2.0	4.0	6.0	8.0	10.0
LPI	232.56 ± 2.97^Bc^	157.56 ± 2.38^Ec^	192.92 ± 2.85^Db^	220.93 ± 4.84^Cc^	258.70 ± 1.73^Ac^
LPH	196.26 ± 2.11^Be^	138.05 ± 2.69^De^	169.59 ± 1.97^Cc^	198.41 ± 3.08^Bd^	228.66 ± 1.91^Ae^
LPHC	208.58 ± 1.74^Cd^	156.75 ± 6.10^Ed^	189.51 ± 1.90^Db^	224.70 ± 3.18^Bc^	237.28 ± 2.07^Ad^
MLPH	274.10 ± 3.01^Ca^	212.37 ± 0.95^Ea^	235.91 ± 4.85^Da^	312.62 ± 2.34^Ba^	369.64 ± 1.80^Aa^
MLPHC	256.10 ± 1.34^Cb^	199.97 ± 0.33^Eb^	231.58 ± 0.92^Da^	267.12 ± 1.70^Bb^	288.22 ± 2.10^Ab^

*Note*: Different small and large superscripts indicate statistically significant difference between the columns and rows, respectively. The samples were prepared at 10 mg/mL.

At high acidic condition such as pH 2.0, EAI and ESI were higher than the values obtained at isoelectric point. When proteins, peptides, and grafted complexes are subjected to the acidic condition, the amino acids mainly get positive charges, thus, electrostatic repulsions between the chains become predominant (Rezvankhah et al., 2020; Rezvankhah, Emam‐Djomeh, et al., 2022). In this state, proteins, peptides, and conjugates act as efficient emulsifiers that can reduce the interface tension, coat the oil droplets, stabilizing them within the continuous phase (Rezvankhah, Yarmand, & Ghanbarzadeh, 2022). As shown in Table 2, the emulsions at pH 2.0 indicated higher ESI, even when compared to emulsions at pH 6.0. Moreover, MLPH indicated the highest EAI and ESI at pH 2.0, which showed that Maillard grafting had improving effects on the functional properties. By shifting pH to 6.0, a slight increase in EAI of LPHC, MLPH, and MLPHC was observed while ESI values were decreased. It could be attributed to the insufficient negative charges that existed on the peptide molecules. By shifting to pH 8.0, EAI and ESI values were significantly increased while the changes were so highlighted for MLPH and MLPHC. Cross‐linking and subsequently conjugation with GA efficiently improved the functional attributes. MTGase‐mediated cross‐linking can polymerize the peptides, increase the MW, and improve the hydrophobic/hydrophilic balance. In this state, the cross‐linked peptides can potentially migrate to the interface and reduce the interfacial tension (Liu, Wang et al., 2022; Rezvankhah, Yarmand, & Ghanbarzadeh, 2022; Rezvankhah et al., 2023). On another side, Maillard conjugation between the peptides and polysaccharides can produce complexes with well‐balanced hydrophobic/hydrophilic properties that can potentially participate in emulsion formation and stabilization (Hou et al., 2017; Seidi et al., 2021).

At high basic condition (pH 10.0), the highest EAI and ESI values were achieved while MLPH indicated the highest values among the samples. At basic condition, the electrostatic repulsion between the peptide chains and also grafted high molecular GA chains is dominated and the oil droplets efficiently are coated by the stabilizer/emulsifier (Zha, Yang, et al., 2019). In this state, the flocculation of oil droplets much lower occurs and emulsions are maintained thermodynamically stable for a long period (Nasirpour & Saeidy, 2022). One of the most important factors in the production of stable emulsions is hydrophobic/hydrophilic balance (Rezvankhah, Yarmand, & Ghanbarzadeh, 2022). Hydrolysis of polypeptides impressively increased the surface hydrophobicity while cross‐linking by MTGase and conjugation with GA had decreasing effects. Thus, in addition to hydrophobicity interactions, hydrophilic interactions are also necessary. In this regard, LPI indicated higher EAI and ESI values compared to LPH at all pH values. When the oil and aqueous phases rich in proteins are mixed and subjected to homogenization, proteins migrate to the oil–water interfaces where they realign themselves to position the surface hydrophobic amino acids toward the oil phase and hydrophilic moieties within the aqueous phase (Liu, Pei, & Heinonen, 2022; Rezvankhah, Emam‐Djomeh, et al., 2022). The migrated proteins form an interfacial film surrounding the oil droplets that maintain stability via electrostatic repulsive forces, steric stabilization, and decreased interfacial tension. LPI has been reported to reduce the interfacial tension by ~50% in canola oil–water mixtures (Liu, Pei, & Heinonen, 2022). The predominant hydrophobic/hydrophobic interactions between LPH and oil droplets led to lower EAI and ESI values. Further modification of LPH through MTGase‐mediated cross‐linking and Maillard conjugation led to improvement of hydrophilic interactions and a well‐balanced hydrophobic/hydrophilic ratio was established. Several studies have confirmed that the Maillard reaction between plant proteins and sugars/polysaccharides has led to improvement of emulsifying properties (Kan et al., 2021; Ke & Li, 2023; Pirestani et al., 2017; Zhao et al., 2022).

### 3.9 Foaming properties

The effects of hydrolysis, cross‐linking, conjugation of hydrolysates with GA, and combined cross‐linking and conjugation were investigated on FC and FS values (Table 3). Hydrolysis of LPI led to reduction of FC although it was insignificant (*p* > .05) while FS was significantly decreased (*p* < .05). MTGase‐mediated cross‐linking of LPH led to significant reduction of FC while FS was not altered. FC is directly related to the interaction of emulsifiers with air and water (Avramenko et al., 2013; Rezvankhah et al., 2023; Rezvankhah, Yarmand, & Ghanbarzadeh, 2022). Cross‐linking reburied the peptide structure and hydrophobic segments were covered (Rezvankhah, Yarmand, & Ghanbarzadeh, 2022). Covering the hydrophobic segments can reduce the aeration capacity (Rezvankhah, Yarmand, & Ghanbarzadeh, 2022). When the complexes of LPH and LPHC and GA were formed, FC and FS were significantly increased, which might be related to well‐balanced hydrophobic–hydrophilic nature of produced emulsifiers (Nooshkam et al., 2020; Rezvankhah, Yarmand, & Ghanbarzadeh, 2022). MLPHC indicated the highest FC and FS values and it was concluded that conjugation had impressive impacts on the improvement of foaming properties. Conjugation of LPH and LPHC with GA led to high foam formation while also delaying the collapsing of the foam (Kan et al., 2021). GA also has a protein moiety that augmented the foam formation (Kan et al., 2021; Rezvankhah, Emam‐Djomeh, et al., 2022).

**TABLE 3:** Effects of hydrolysis of LPI, cross‐linking of hydrolysates, and conjugation of hydrolysates with gum Arabic, and combined cross‐linking and Maillard conjugation of hydrolysates with gum Arabic.

Sample	Foaming capacity (%)	Foaming stability (%)
LPI	80.10 ± 2.10^c^	57.89 ± 0.20^c^
LPH	76.47 ± 1.50^c^	23.53 ± 0.90^d^
LPHC	41.18 ± 2.10^d^	23.53 ± 2.30^d^
MLPH	88.57 ± 3.10^b^	60.57 ± 0.50^b^
MLPHC	142.86 ± 3.60^a^	72.00 ± 2.60^a^

*Note*: Different small letters in each column indicate statistically significant difference (*p* < .05). Samples were prepared at 10 mg/mL.

### 3.10 Sensory properties

**FIGURE 6:**
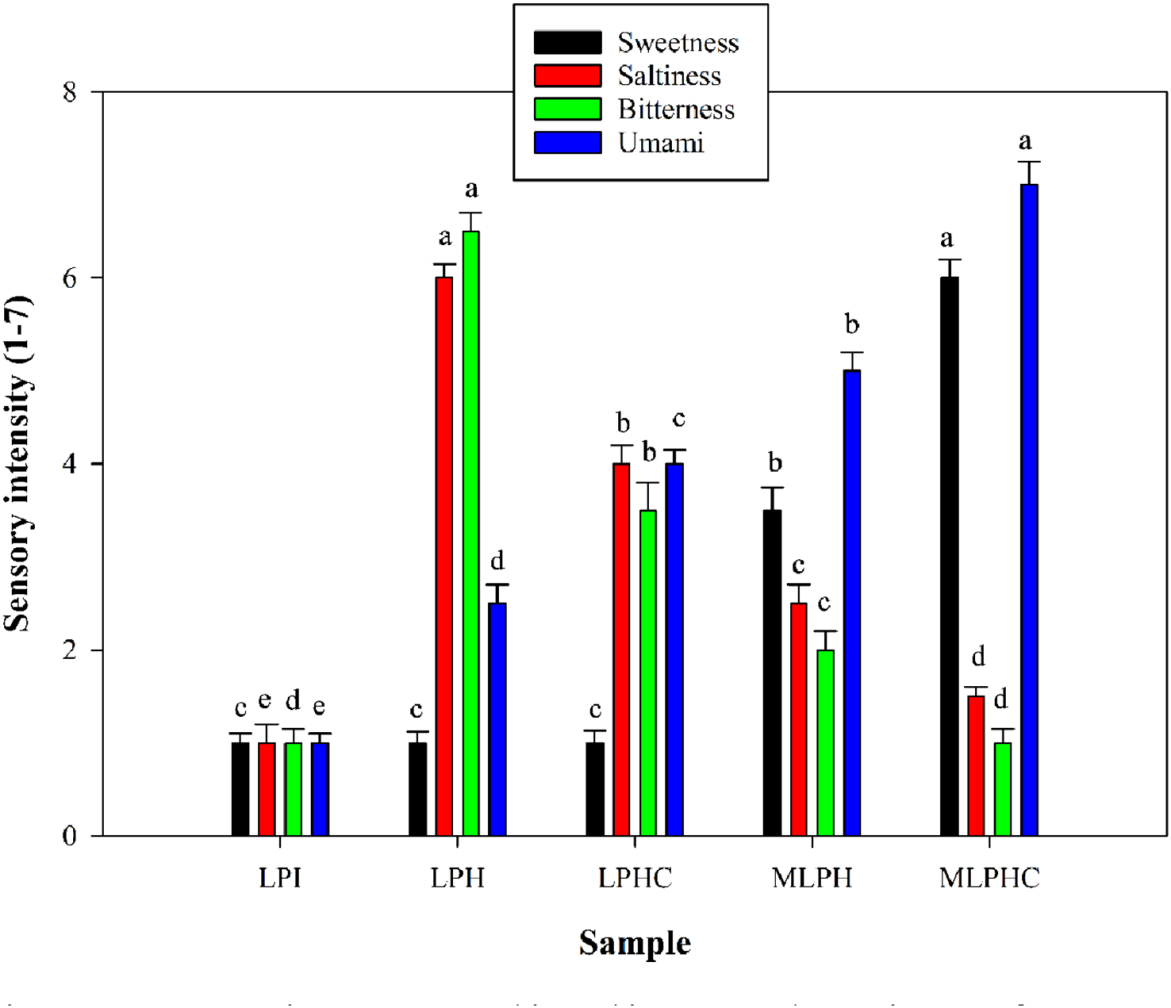
Sensory properties (sweetness, saltiness, bitterness, and umami tastes) of LPI, LPH, LPHC, MLPH, and MLPHC. The effects of hydrolysis of LPI, cross‐linking of LPH, and combined cross‐linking and Maillard reaction between LPH and LPHC with gum Arabic were investigated. The samples were scored from 1 to 7 based on 7‐point hedonic test.

The sensory properties including sweetness, saltiness, bitterness, and umami were determined according to Figure 6. Hydrolysis significantly increased the saltiness, bitterness, and umami tastes (*p* < .05). Hydrolysis could break down the peptide chains, liberating peptides with low MW, and exposing the amino acids contributing predominantly to umami taste (Rezvankhah et al., 2021a, 2023). Cross‐linking of LPH by MTGase led to reduction of saltiness and bitterness while umami taste was increased (*p* < .05). These results were in agreement with those achieved in previous studies (Rezvankhah et al., 2021a; Song et al., 2013). Cross‐linking can accumulate the peptides with amino acids that predominantly contribute to the umami taste (Rezvankhah, Yarmand, & Ghanbarzadeh, 2022; Song et al., 2013). On the other hand, conjugation of LPH with GA led to more reduction of saltiness and bitterness while sweetness and umami tastes were increased. In this regard, MLPHC indicated the highest sweetness and umami tastes conception while the saltiness and bitterness reached the lowest tastes conception. Several studies have reported that Maillard reaction can lead to the formation of conjugates along with modified flavor while enhanced umami taste and meaty‐analog flavor were certified (Habinshuti et al., 2019; Song et al., 2013; Wei et al., 2018; Zha, Dong, et al., 2019). The sensory results of the present study were also in agreement with the changes in volatile compounds that occurred after hydrolysis, cross‐linking, and predominantly after conjugation and combined cross‐linking and conjugation. Hydrolysis solely has shown to be positively effective on the development of umami taste. Cross‐linking has also shown to accumulate the umami amino acids and also increase the meaty flavor. Maillard reaction between the hydrolysates and polysaccharides can more efficiently promote the umami taste not only due to the presence of umami amino acids, but also due to the generation of volatile components or changes of inherent volatiles.

In our previous study, sequential hydrolysis and cross‐linking of LPH led to the development of umami taste (Rezvankhah et al., 2023). In addition, development of umami taste in MLPH and MLPHC increased their chance to be used in meat products as a replacer of meat proteins and flavor enhancers such as MSG with high health risks (Rezvankhah et al., 2023; Zanfirescu et al., 2019).

## 4 CONCLUSIONS

The effects of hydrolysis, cross‐linking, and mainly Maillard grafting of hydrolysates with GA were investigated. The hydrolysis increased the antioxidant activity and surface hydrophobicity, while the cross‐linking and especially Maillard reaction decreased them. Hence, MLPH and MLPHC indicated the lowest surface hydrophobicity and antioxidant activity. Maillard grafting between the hydrolysates and GA led to the formation of high MW bands (>250 kDa). The FTIR analysis indicated that amide I and amide II structures were altered after Maillard modification. The inherent volatile compounds, such as alcohols, ketones, aldehydes, and acids, were changed after hydrolysis, cross‐linking, and predominantly Maillard reaction, reducing the grassy and plant tastes. Maillard grafting led to improvement of functional properties, while MLPH and MLPHC indicated the highest EAI, ESI, FC, and FS. Sensory analysis exhibited that umami taste was highly developed in MLPH and MLPHC, which caused high potential application of them to be used in meat products as a replacer of meat protein and flavor enhancers such as MSG.

## AUTHOR CONTRIBUTIONS

**Amir Rezvankhah:** Conceptualization (lead); data curation (lead); formal analysis (lead); funding acquisition (lead); investigation (lead); methodology (lead); project administration (lead); resources (lead); software (lead); supervision (lead); validation (lead); visualization (lead); writing – original draft (lead); writing – review and editing (lead). **Babak Ghanbarzadeh:** Conceptualization (equal); formal analysis (equal); investigation (equal); methodology (equal); project administration (equal); supervision (equal); validation (equal); visualization (equal). **Homaira Mirzaee:** Conceptualization (equal); data curation (equal); investigation (equal); methodology (equal); writing – review and editing (equal). **Ali Ahmadi Hassan Abad:** Funding acquisition (equal); investigation (equal); methodology (equal). **Ali Tavakkoli:** Funding acquisition (equal); investigation (equal); methodology (equal). **Alireza Yarmand:** Investigation (equal).

## FUNDING INFORMATION

This research study was done by financial supports of Dr. Rezvankhah, Dr. Ghanbarzadeh, Mr. Ahmadi Hassan Abad, and Mr. Tavakkoli.

## CONFLICT OF INTEREST STATEMENT

None of authors declare conflict of interest.

## Data Availability

The data supporting the results of this study were provided in the form of tables and figures. All authors state that additional data will be made available upon request to the corresponding author.
